# A youth-led social marketing intervention to encourage healthy lifestyles, the EYTO (European Youth Tackling Obesity) project: a cluster randomised controlled0 trial in Catalonia, Spain

**DOI:** 10.1186/s12889-015-1920-1

**Published:** 2015-07-03

**Authors:** Elisabet Llauradó, Magaly Aceves-Martins, Lucia Tarro, Ignasi Papell-Garcia, Francesc Puiggròs, Lluís Arola, Jordi Prades-Tena, Marta Montagut, Carlota M Moragas-Fernández, Rosa Solà, Montse Giralt

**Affiliations:** Health Education and Promotion, NFOC Group, Facultat de Medicina i Ciències de la Salut, Universitat Rovira i Virgili, Reus, Spain; Centre Tecnològic de Nutrició i Salut (CTNS), TECNIO, CEICS , Avinguda Universitat, 1, Reus, 43204 Spain; Departament de Bioquímica i Biotecnologia, Nutrigenomics Research Group, Universitat Rovira i Virgili, Tarragona, Spain; Departamento de Estudios de Comunicación, Universitat Rovira i Virgili, Tarragona, Spain; Unit of Lipids and Arteriosclerosis Research, CIBERDEM, NFOC group, Facultat de Medicina i Ciències de la Salut, Hospital Universitari Sant Joan, IISPV, Universitat Rovira i Virgili, C/Sant Llorenç 21, Reus, 43201 Spain; Unit of Pharmacology, NFOC group, Facultat de Medicina i Ciències de la Salut, Universitat Rovira i Virgili, Reus, Spain

**Keywords:** Adolescents, Youth, Peer-led, Healthy lifestyles, Obesity, Social marketing, Study protocol

## Abstract

**Background:**

The encouragement of healthy lifestyles for obesity prevention in young people is a public health priority. The European Youth Tackling Obesity (EYTO) project is a multicentric intervention project with participation from the United Kingdom, Portugal, the Czech Republic and Spain. The general aim of the EYTO project is to improve lifestyles, including nutritional habits and physical activity practice, and to prevent obesity in socioeconomically disadvantaged and vulnerable adolescents. The EYTO project works through a peer-led social marketing intervention that is designed and implemented by the adolescents of each participating country. Each country involved in the project acts independently. This paper describes the “Som la Pera” intervention Spanish study that is part of the EYTO project.

**Methods/Design:**

In Spain, the research team performed a cluster randomised controlled intervention over 2 academic years (2013–2015) in which 2 high-schools were designated as the control group and 2 high-schools were designated as the intervention group, with a minimum of 121 schoolchildren per group.

From the intervention group, 5 adolescents with leadership characteristics, called “Adolescent Challenge Creators” (ACCs), were recruited. These 5 ACCs received an initial 4 h training session about social marketing principles and healthy lifestyle theory, followed by 24 sessions (1.30 h/session) divided in two academic years to design and implement activities presented as challenges to encourage healthy lifestyles among their peers, the approximately 180–200 high-school students in the intervention group. During the design of the intervention, it was essential that the ACCs used the 8 social marketing criteria (customer orientation, behaviour, theory, insight, exchange, competition, segmentation and methods mix). The expected primary outcomes from the Spanish intervention will be as follows: increases in the consumption of fruits and vegetables and physical activity practice along with reductions in TV/computer/game console use. The secondary outcomes will be as follows: increased breakfast consumption, engagement with local recreation and reduced obesity prevalence. The outcomes will be measured by the Health Behaviour in School-aged Children Study (HBSC) survey at baseline and at the end of the intervention.

In the control group, no intervention was implemented, but the outcome measurements were collected in parallel with the intervention group.

**Discussion:**

This study described a new methodology to improve lifestyles and to address adolescent obesity.

**Trial registration:**

ClinicalTrials.gov: NCT02157402. Registered 03 June 2014.

**Electronic supplementary material:**

The online version of this article (doi:10.1186/s12889-015-1920-1) contains supplementary material, which is available to authorized users.

## Background

Obesity is an important global public health problem, and its long-term consequences are well documented [1–3]. The prevalence of excess weight and obesity in childhood is increasing in different countries. In Europe, the prevalence of excess weight, including overweight and obesity, is 19–49 % in boys and 18–43 % in girls, whereas the obesity prevalence is 6–26.6 % in boys and 5–17 % in girls [4]. In developed countries, obesity is also related to socioeconomic status: obesity rates follow a social gradient in which the highest rates are present in racial/ethnic minorities and socioeconomically disadvantaged populations [5, 6].

Once obesity is established, it is difficult to treat, highlighting the urgent need for successful strategies and policies to revert trends in weight gain, sedentary lifestyles and inadequate nutritional habits, especially in vulnerable youth populations [6–8].

The improvement of healthy lifestyles through modification of eating habits, daily physical activity practice and avoiding sedentary behaviour are the principle modifications that can prevent or reduce the risk of obesity [9]. Specific recommendations based on expert opinion or supported by clinical studies are proposed [10]. These recommendations are the periodic surveillance of obesity status of children and adults, education of children and families about healthy lifestyles, community enrolment in health advice and health education, assure a balanced nutrition and breastfeeding in early infancy and perinatal period, school-based interventions on health education focused on healthy eating and physical activity, home-based interventions, and support of health authority and registration. The authorities should contribute in encouraging people in disadvantaged areas to eat healthier by improving the availability, quality and pricing of healthy food in these localities [11] and encouraging them to perform more physical activity by providing access to sport grounds and green spaces [12].

Nutrition and healthy lifestyle education for adolescents have to be planned differently than for other educational ages because the cognitive and social developmental processes, such as the shifts towards abstract thinking and problem-solving skills, questioning adult authority, increased autonomy from parents, and an increased reliance on peers as a source of identity, support, and normative behaviour, develop during this life period [13–20]. This age period presents both a challenge and an opportunity to offer new learning and teaching strategies to engage adolescents and motivate them to make healthy food choices [13, 14]. The influence of peers on young people’s health behaviours is acknowledged [16–19], and interventions using a peer-led model for health promotion have shown positive effects [20].

Social marketing when it is conscientiously applied, has been identified as a possible strategy to change behaviours [21]. Kotler and Zaltman expressly defined social marketing in 1971 as “a social influence technology involving the design, implementation and control of programs aimed at increasing the acceptability of a social idea or practice in one or more groups of target adopters” [22]. This term was re-described by Andreasen in 1994 as “the application of commercial marketing technologies to the analysis, planning, execution and evaluation of programs designed to influence the voluntary behaviour of target audiences in order to improve their personal welfare and that of their society” [23].

Doctrines and tactics from commercial marketing for social change programs can improve the strategic value of health communication and increase the likelihood that people will make healthy behavioural choices [22–25].

To help strengthen the use of effective social marketing approaches, the Social Marketing National Benchmark Criteria (SMBC) was developed by the National Social Marketing Centre (NSMC) in the United Kingdom [26]. The purpose of this benchmark is to create support for a better understanding of social marketing that takes into account the 8 basic SMBC principles: customer or participant orientation, behaviour, theory, insight, exchange, competition, segmentation and methods mix, as well as the promotion of a consistent approach to review and evaluate projects [26].

Some studies suggest that the use of social marketing strategies to modify behaviour, lifestyles and other aspects of diet and physical activity through an intervention (target audience played an active role) or a campaign (target audience played a passive role) can reduce the overweight or obesity prevalence among children and adolescents. There are some social marketing campaigns that demonstrate positive attitude and behaviour effects in children, such as the VERB social marketing campaign to increase physical activity among youth [27], Canada’s ParticipACTION national physical activity mass media campaign targeting parents of elementary school-aged children [28] and an intervention focused on improving the snacking habits of pre-school children [29]. By contrast, “The 5,4,3,2,1 go! Intervention” [30] demonstrated effects on parental behaviour and did not affect children [31]. The Change4Life campaign, a national social marketing program implemented in the United Kingdom to reduce obesity [32], demonstrated positive effects on awareness but little impact on attitudes and behaviours. However, the effectiveness of these campaigns requires further research on behaviour modification using randomised, controlled intervention studies to determine the appropriate number of criteria and the key social marketing criteria that will have the greatest impact on achieving the intervention objectives.

The European Youth Tackling Obesity (EYTO) project looks to contribute the description and evaluation of interventions to tackle obesity in adolescents by recruiting young people to design and implement peer-led social marketing interventions that promote healthy eating and physical activity among young people aged 13–16 years with low income who are vulnerable to obesity. The general aims of the EYTO project are to improve lifestyles, such as nutritional habits and physical activity practice, and to prevent obesity in socioeconomically disadvantaged and vulnerable adolescents. The secondary aims are to reduce the modifiable causes of obesity amongst disadvantaged young people; to improve health, education and social outcomes for young people who are obese; to contribute to a reduction in health inequalities among young people; and to increase the participation of young people in the development of interventions to address obesity.

This paper describes the “Som la Pera” Spanish intervention study design of the EYTO project, which proposed performing a school cluster randomised controlled trial.

## Methods

### European study design

This is a multicentric peer leadership intervention involving the participation of the United Kingdom, Spain, Portugal and the Czech Republic. Five selected adolescents (in each country) are tasked with designing and implementing a social marketing intervention, using the SMBC as a basis for the design, for 180–200 schoolmates. The selection of 5 ACCs per country was done as an easy and rapid way to design and prepare the challenges. The intervention should encourage healthy lifestyles among their peers of the same age in disadvantaged neighbourhoods; the adolescent peer-led model is more effective at achieving positive results in health behaviour than the adult-led models applied in school-based studies [33]. Adolescent peer-led interventions use the youth empowerment theory based on engaging young people in the decision-making process to improve their health and well-being [34]. Because the EYTO is a multicentric project in which each country acts autonomously and because the design and implementation of the interventions are directed by different adolescents in the participating countries, this protocol only reports the description of the Spanish study design.

The procedures and progress reports of work deliverables will be led by the Spanish management team according to the schedule’s National Children’s Bureau (NCB) to the European Commission, with a frequency of 6 months.

The communication of the results to the participants, healthcare professionals, and the public will be performed via publication, reporting in a results database, and other data-sharing arrangements. Authorship eligibility is in accordance with the best practices and ethical guidelines. If necessary, we will guarantee public access to the full protocol, participant database and statistical code.

### Spanish study design

**Fig. 1:**
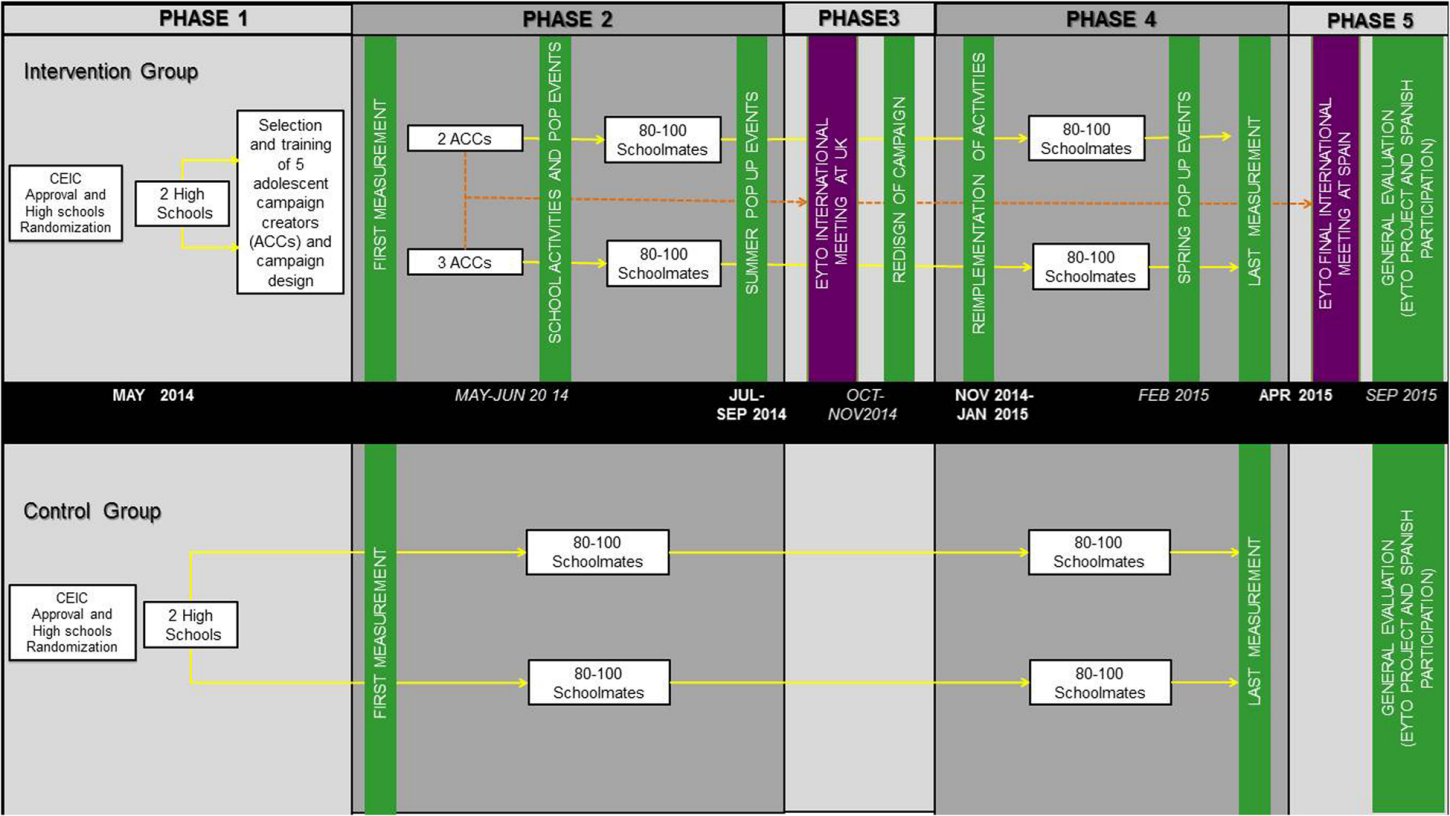
Spanish Intervention Schedule of the European Youth Tackling Obesity (EYTO) Project

The “Som la Pera” intervention is a school cluster randomised controlled trial. The participating Spanish city was Reus (Catalonia). Local authorities have already identified public high-schools that they agree serve low-income neighbourhoods that are considered disadvantaged areas. From the 9 public high-schools identified in these neighbourhoods, 4 high-schools were randomly selected. The randomisation code was computer generated. The high-schools were assigned to the control or intervention arm at a ratio of 1:1 via an interactive electronic response system hosted by the Nutrition and Health Technology Centre (CTNS) in Reus, Spain. The unit responsible for the randomisation took no further part in the study. Because the researchers know the names of the four high-schools, allocation concealment was not performed. The project consists of five main phases, as shown in Fig. 1. The first phase included the randomisation and allocation of the high-schools in the disadvantaged area into the two intervention and two control high-schools. High-school teachers from the randomised intervention group selected the five adolescents according to their knowledge of the students by considering leadership characteristics and English level (because the students will participate in EYTO European meetings), 2 adolescents from one high-school and 3 from the other high-school. These 5 adolescents will be referred to in this protocol as the Adolescent Challenge Creators (ACCs). The study characteristics are summarised in Table 1.

**Table 1:** General study characteristics

Arm	High school information	Assigned interventions	Population	Primary and secondary outcomes
Intervention group	High-school A	The intervention group will receive an intervention consisting of challenges designed by 5 ACCs. These activities must have social marketing criteria.	High-school A	Primary outcome: consumption of fruits and vegetables, physical activity practice, and TV/computer/game console use. Secondary outcomes: breakfast consumption, engagement with local recreation and obesity prevalence
The intervention group received challenges designed by ACCs that promote healthy lifestyles.	Economically disadvantaged		Students from high schools from low-income neighbourhoods, who are 13 to 16 years of age:	
	Size: 3 classes/level		a) 3 to 5 ACCs	
	Public		b) 80-100 adolescents	
	Reus			
	High-school B		High-school B	
	Economically disadvantaged		Students from high schools from low-income neighbourhoods, 2who are 13 to 16 years of age:	
	Size: 4 classes/level		a) 2 to 5 ACCs	
	Public		b) 80-100 adolescents	
	Reus			
Control group	High-school A	No intervention is assigned for this group.	High-school A	The same outcomes were measured with the same tools and over the same time frame as in the intervention group.
The control group no received challenges to promote healthy lifestyles.	Economically disadvantaged		Students from high schools from low-income neighbourhoods, who are 13 to 16 years of age:	
	Size: 3 classes/level		b) 80-100 adolescents	
	Public			
	Reus			
	High-school B		High-school B	
	Economically disadvantaged		Students from high schools from low-income neighbourhoods, who are 13 to 16 years of age:	
	Size: 4 classes/level		b) 80-100 adolescents	
	Public			
	Reus			

### Participants and professional experts

The inclusion criteria were as follows: participants were between 13 and 16 years of age, attended one of the four randomised high-schools and had an informed consent signed by their parents or legal guardians. In addition, the five ACCs selected in the intervention group were included if they fulfilled the inclusion criteria mentioned previously, displayed leadership characteristics, had at least a working knowledge of the English language (for the International EYTO meetings) and were highly motivated and committed to the study. The lack of any inclusion criteria was the first and only exclusion criterion.

The professional experts who participate in “Som la Pera” intervention are:

- Physicians: physicians specialist on health education and promotion led the implementation of the study from the recruitment process to the end of the experimental protocol by meetings with stakeholders like high-schools’ director or local policy-makers and health and educators administrators. They were in charge of designing, performing and revising the evaluation process throughout the study.
- Nutritionists: nutritionists with expertise on health education and health promotion led the high-school recruitment process by meetings with parents and adolescents explaining the project, recollected the informed consent of parents and adolescent to participate in the study and, coordinated the logistical issues of the study. They contributed to support the evaluation of primary and secondary outcomes through the validated questionnaire in high-schools computer classrooms to solve adolescents’ questions of lifestyles and behaviour evaluation questionnaire. The nutritionists were responsible for the dietary and healthy lifestyle training of the 5 ACCs.
- Managers: The managers coordinated the Spanish intervention among the participating countries. They supervised all of the scientific work, from the intervention trial design to the scientific data interpretation.
- Publicists and Journalists: publicists and journalists experts on health communication were responsible for the communication training of the 5 ACCs. Even though the 5 ACCs were in charge of the communication and dissemination campaign (lifestyles messages and intervention challenges), publicists and journalists are in charge to disseminate the general information of this intervention and European project to general population through local, national and international newspapers and television media.

### Intervention

- Training process

The 5 ACCs received a 4 h initial training session about social marketing principles and the healthy lifestyle theory from a university specialist in health and communication. Moreover, the 5 ACCs received 1.30 h of training every week over 12 weeks (1st academic year) and 1.30 h of training every week over 12 weeks (2nd academic year), a total of 12 sessions/academic year, leading to a total of 24 sessions (1.30 h/session), also performed by a university specialist. The aims of the sessions were to train the 5 ACCs on health promotion, health education, communication and social media so that they could design the challenge activities for their peers. The university specialists educated the 5 ACCs about the primary and secondary objectives of the intervention, and the ACCs then had to design the challenges for their schoolmates to accomplish the defined objectives.

The 5 ACCs designed social marketing activities proposed as challenges based on 8 SMBCs: 1) customer orientation, 2) behaviour, 3) theory, 4) insight, 5) exchange, 6) competition, 7) segmentation and 8) methods mix (Table 2). These 5 ACCs were recruited separately from two different high-schools. Then, the 5 ACCs, as peer-led instructors, identified the possible lifestyle components that they and their peers should improve, selected the easiest and most common communication channels among them, and determined which challenges should be designed and implemented for their peers.

**Table 2:** Use of SM in the intervention

Research group	Adolescent Challenge Creators (ACCs)	Research group and Adolescent Challenge Creators (ACCs)
*1. Customer Orientation*: Focuses on the audience to help understand their lives, behaviours and issues.	*2. Behaviour*: Aims to change people’s actual behaviours.	3.*Theory*: Uses behavioural theories to understand behaviour and to inform the intervention.
The peer-led model attracts the motivation of adolescents to participate and interact in the intervention, because adolescents prepare activities directed to adolescents. In this way, it has in mind their motivations and behaviours.	Aims to improve the consumption of fruits and vegetables, PA practice, and breakfast consumption and decrease the TV, PC and video game behaviour.	It used the behavioural change framework, taking into account the “Behaviour Change Wheel” (Michie, van Stralen, & West, 2011).
5. *Exchange*: Considers the benefits and costs of adopting and maintaining a new behaviour. The perceived cost can be social, economic, or physical.	4. *Insight*: develop a deep understanding of the target audience.	6. *Competition*: Seeks to understand the possible barriers for the audience’s time, attention, and inclination to behave in a particular way.
The consideration of the cost-effectiveness of the intervention will be evaluated at the end-of-intervention.	The peer-led model motivates adolescents to participate and interact in the intervention because adolescents prepare activities directed towards adolescents. In this way, it has in mind their motivations and behaviours.	The 5 adolescent coordinators discussed the enablers and barriers that adolescents face when making behavioural changes. From this debate, some changes were proposed to facilitate the process by including stakeholders.
*8. Methods Mix*: Uses a mix of methods to bring about behavioural change. Does not rely solely on raising awareness.		7. *Segmentation*: Identifies audience “segments” that have common characteristics and then tailors interventions appropriately.
It contributed to informing using social media, educating using activities designed by adolescent coordinators and social media, and supporting using visual material in high-schools and social media. The design and control will be applied using the suggestions provided by 5 adolescent coordinators.		The intervention is focused on adolescents 13 to 16 years of age who attend the participant high-schools and are from low socioeconomic status neighbourhoods.

Social Marketing National Benchmark Criteria (SMBC) developed by the National Social Marketing Centre (NSMC) UK [23]

b) Design and implementation process

The ACCs chose the name “Som la Pera” for the Spanish intervention. This name is a Catalan idiom that literally means “we are a pear” but figuratively means “we are cool”. The ACCs designed and implemented the following challenges: gymkhanas (an activity inside high-schools in which adolescents were divided by teams and competed among themselves in different sport and food tasting competitions, such as goal scoring, racing, or discerning foods with one’s eyes closed) and cooking ability and lifestyle knowledge competitions (high-school cooking competition to prepare healthy dishes simulating cooking TV show or quiz show), as well as pop-up events that included healthy cooking contests and lifestyle knowledge competitions. The 5 ACCs had to be in touch with community stakeholders to obtain some resources free of charge. For example, Central Mercat de Reus gave them food to develop cooking competitions and run the gymkhana, and local government provided local community spaces to develop challenges. Moreover, material costs for items such as posters, flyers, etc. were paid for by the project budget. The ACCs chose Facebook as the main channel of communication with their peers (https://www.facebook.com/somlapera). The intervention designed by the Spanish ACCs was launched in Reus, Spain, in May 2014.

The 5 challenges designed by the ACCs over the first year in the intervention high-schools were implemented for a period of 12 weeks (second phase Fig. 1). At the end of this phase, there was an EYTO project meeting in London (UK) at which the 5 ACCs from the 4 participating countries came together to exchange experiences and ideas so they could revise, maintain or add new components to their second-year design intervention. The costs of this meeting were included in the project funding (third phase Fig. 1). Five new challenges designed by the ACCs were implemented during the second academic year, for a period of 12 weeks (fourth phase Fig. 1). And finally, last European EYTO meeting in Spain to conclude the participation of the ACCs were done to exchange intervention experiences across participating countries (fifth phase Fig. 1).

In the control group, no intervention will be implemented, but the outcome measurements were collected in parallel with the intervention group.

### Evaluation process

#### Primary and secondary outcomes

The expected primary outcomes of the Spanish intervention were as follows: increases in the consumption of fruits and vegetables and physical activity practice, along with reductions in TV/computer/game console use. The secondary outcomes were as follows: increased breakfast consumption, engagement with local recreation and reduced obesity prevalence. The outcomes of the Spanish intervention were measured by the Health Behaviour in School-aged Children Study (HBSC) Survey 2009–2010 [35] to evaluate the adolescents’ lifestyles at baseline and at the end of the intervention as presented in Table 3. The HBSC study is a validated cross-sectional survey of school students that collects data every four years on 11-, 13- and 15-year-old adolescents [36]. The baseline measurements from the intervention and control groups were performed in the second phase, and the end-of-study measurements were performed in the fourth phase (Fig. 1). Analyses and evaluations of each country’s intervention and global project will be performed in phase 5 (Fig. 1) so that the project can be concluded.

**Table 3:** Outcomes measurements in the Health Behaviour in School-aged Children Study (HBSC) Survey

Health Behaviour in School-aged Children Study Items	Outcome measured	Question	Possible answers
Eating habits	Nutritional behaviour. Fruit, vegetable and water consumption.	How many times a week do you usually eat or drink (fruits, vegetables, sweets, coke or other soft drinks that contain sugar)…?	Never, less than once a week, once a week, 2–4 days a week, 5–6 days a week, once a day, every day, every day more than once every day.
(primary outcomes)	Breakfast quantity and quality	How often do you usually have breakfast (more than a glass of milk or fruit juice)?	*Weekdays* (I never have breakfast, one day, two days, three days, four days, five days).
			*Weekends* (I never have breakfast during the weekend, I usually have breakfast on only one day of the weekend (Saturday OR Sunday), I usually have breakfast on both days (Saturday AND Sunday)).
Physical activity	Physical activity practice	Over the past 7 days, on how many days were you physically active for a total of at least 60 min per day?	0 days, 1 day, 2 days, 3 days, 4 days, 5 days, 6 days, 7 days.
(primary outcomes)			
		Outside school hours: How often do you usually exercise in your free time so much that you get out of breath or sweat?	Every day, 4–6 times a week, 2–3 times a week, once a week, once a month, less than once a month, never.
		Outside school hours: How many hours a week do you usually exercise in your free time so much that you get out of breath or sweat?	None, about half an hour, about 1 h, about 2 to 3 h, about 4 to 6 h, about 7 h or more.
Sedentary behaviour	Sedentary behaviour	About how many hours a day you usually watch television (includes DVD and videos) in your free time?	Weekdays: None at all, about half an hour a day, about 1 h a day, about 2 h a day, about 3 h a day, about 4 h a day, about 5 h a day, about 6 h a day, about 7 or more hours a day.
(primary outcomes)			Weekends: None at all, about half an hour a day, about 1 h a day, about 2 h a day, about 3 h a day, about 4 h a day, about 5 h a day, about 6 h a day, about 7 or more hours a day.
		About how many hours a day do you usually play games on a computer or games console (PlayStation, Xbox, GameCube, etc.) in your free time?	Weekdays: None at all, about half an hour a day, about 1 h a day, about 2 h a day, about 3 h a day, about 4 h a day, about 5 h a day, about 6 h a day, about 7 or more hours a day.
		About how many hours a day do you usually use a computer for chatting on-line, internet, emailing, homework, etc. in your free time?	Weekends: None at all, about half an hour a day, about 1 h a day, about 2 h a day, about 3 h a day, about 4 h a day, about 5 h a day, about 6 h a day, about 7 or more hours a day.
Self-confidence	Weight control and body image	At the present, are you on a diet or doing something else to lose weight?	No, my weight is fine; No, but I should lose some weight; No, because I need to put on weight; Yes.
(secondary outcomes)			
Body mass index (secondary outcomes)	Perceived obesity and overweight prevalence	How much do you weigh without clothes?	Free space for answer.
		How tall are you without shoes?	Free space for answer.

Items obtained from the Health Behaviour in School-Aged Children (HBSC) 2009/2010 Ref. [35]

#### Statistical analysis plan

We estimated that with a sample size of 121 adolescent schoolmates per group, the study will have 90 % power to detect a difference of a 0.5 portion of vegetables or fruits between the intervention and control groups, setting the bilateral level of statistical significance at 5 %. We anticipate a loss of follow-up rate of 30 %.

Analyses of the results will be performed on an intent-to-treat (ITT) basis, defined as a participant who had at least baseline efficacy data.

The descriptive results are expressed as the means ± standard deviations and the 95 % confidence intervals for quantitative variables or as frequency distributions for qualitative variables. Generalised linear mixed models are used to analyse differences between the intervention and control groups and changes in primary and secondary outcomes from baseline to the end of the intervention. For the rest of the efficacy variables, we will use Fisher’s exact test for the categorical variables and Student’s *t*-test for the continuous variables. The significance level is fixed at a bilateral level of 5 %.

All statistical analyses are performed with SPSS version 22.0 (SPSS, Inc., IBM, Armonk, NY, USA).

#### Ethical approval and trial registration

The study has the approval of the Ethical Committee of the Hospital Universitari Sant Joan de Reus (ref: 14–04–24/4proj2), and the trial was registered with clinicaltrials.gov (NCT02157402).

The protocol is in accordance with the Helsinki Declaration and the good clinical practice guidelines of the International Conference of Harmonization (ICH GCP). This randomised trial was conducted according to the extended cluster CONSORT 2010 guidelines.

#### Additional information

This study followed the SPIRIT [37] and TIDieR protocol description recommendations [38] as presented in Additional file 1 and Additional file 2. The participation, schedule of enrolment, interventions and assessment are presented in Table 4. Finally, a cluster CONSORT checklist and flow diagram were used to summarise the description of study when the study and analysis were completed [39].

**Table 4:** Spanish EYTO participation, schedule of enrolment, interventions and assessment

	Study period							
	Enrolment	Allocation	Post-allocation					
	Phase 1				Phase 2			Phase 3
Timepoint	-t_1_	a0	t_1_	t_2_	t_3_	t_4_	t_5_	t_6_
	May 2014_(after CEIC approval)_	May 2014	May 2014	May 2014	May 2014	September 2014	September 2014	October 2014
	High-Schools of low income in Reus are included in this phase.	4 high-school are randomly chosen, 2 in the intervention and 2 in the control group	Control High-school meeting, Separate Intervention high-school meeting and 5 ACC selection	Social marketing, health promotion and communication training for 5 ACCs	Each participant answered the HBSC Survey.	Implementation of challenges by the 5 ACCs with help of stakeholders for their peers.	Preparation of the ACC presentation of the first academic Spanish intervention at the EYTO meeting in London.	Meeting of 5 ACCs from the 4 EYTO participating countries in London to pool designed activities and exchange ideas. Successful intervention activities can be re-implemented.
	Randomisation is performed.			Design of the intervention. Information and informed consent signed	Implementation of interventions by the 5 ACCs for their peers in high-school for 12 weeks.			
Enrolment:	360–400 adolescents from low-income neighbourhoods.							
Eligibility screen	X		X					
Informed consent								
[List other procedures]								
Allocation	180–200 from intervention group and 180–200 from control group							
Intervention			Training of 5 ACCs to design activities		HBSC Survey Implementation of activities during 12-week period	Implementation of social event challenges in the community and local markets for their peers		Evaluate the activities performed during 12-week period (1^st^ academic year)
Control			This group did not receive any interventions		HBSC Survey			
Assessments: List baseline variables	X	X	X				X	X
List primary and secondary outcome variables for 200 participants in the intervention and 200 participants in the control group			Fruit and vegetable consumption, breakfast consumption, physical activity practice and sedentary behaviours.					
Study period								
	Post-allocation					Close-out September 2015		
	Phase 4			Phase 5				
Timepoint	t_7_	t_8_	t_9_	t_10_	t_11_	t_12_		
	November 2014	February 2015	Mar 2015	Sept 2015	Sept 2015	Sept 2015		
	Activities re-implemented by 5 ACCs for their peers in the intervention group high-schools during a 12-week period (2^nd^ academic year)	Challenges designed by the 5 ACCs for their peers with stakeholder help	Preparation of the presentation on the Spanish intervention for the EYTO meeting in Spain	Meeting of 5 ACCs from the 4 EYTO participating countries in Reus, Spain.	Report of intervention challenges to the 4 EYTO countries and web presentation and analysis of data.	EYTO Final report including intervention challenges and Results of lifestyle outcomes		
Interventions:								
Intervention	Implementation of activities during a 12-week period (2^nd^ academic year)	HBSC Survey Intervention implementation						
Control		HBSC Survey						
Assessments:			X	X				
List primary and secondary outcome variables								
for 200 participants in the intervention and								
200 participants in the control group		February 2015, at the end of intervention		End of the participation of the 5 ACCs	Fruit and vegetable consumption.			
					Physical activity Practice. Sedentary behaviours.			

## Discussion

This paper describes an intervention aimed at improving lifestyles, such as nutritional habits and physical activity practice, for obesity prevention in socioeconomically disadvantaged and vulnerable adolescents.

Based on the worldwide current high rates of childhood and adolescent obesity [9], the fight against adolescent OB is a significant public health objective. This focus indicates the necessity of new methodologies to achieve adolescent behaviour changes [40]. Social marketing nutrition interventions were strongly and equally effective at influencing nutrition behaviour, knowledge and psychosocial variables, suggesting that social marketing interventions can produce changes across different behaviours [41]. There is growing evidence that interventions using social marketing approaches can contribute to encouraging healthier lifestyles and, as a result, can prevent obesity [21].

An important problem in the field of social marketing research is that there are some interventions that use social marketing principles in their study design without being aware of that fact [42]. Additionally, few interventions that use the principles consciously publish the obtained results. One such intervention is Change4Life, a national social marketing program, implemented in the United Kingdom to reduce obesity [35]. This intervention achieved increased awareness of the anti-obesity campaign but had little impact on attitudes and behaviours. The peer-led model is effective when it is applied in school-based studies and generates more positive results in health behaviour than adult-led instruction [33]. Also, key principles to create new approaches that fight obesity are set to guide the development of strategies to address unhealthy diets and physical inactivity, and should include: best available scientific evidence, comprehensiveness, multisectoral and multidisciplinary approaches, a life course perspective, addressing poverty, gender and culture sensitivities, and the accountability of all stakeholders to achieve success [43].

Despite their considerable complexity, it is crucial to assess the outcomes achieved in interventions that employ social marketing principles. In this way, the public health sector of the government will be able to distribute its efforts to address adolescent obesity more efficiently [40].

## Additional files

Additional file 1:
**The TIDieR (Template for Intervention Description and Replication) Checklist.**
Additional file 2:
**Standard Protocol Items: Recommendations for interventional trials (SPIRIT) 2013 Checklist.**

## Abbreviations

EYTO: European Youth Tackling Obesity
ACCs: Adolescent Challenge Creators
HBSC: Health Behaviour in School-aged Children Study
SMBC: Social Marketing National Benchmark Criteria
